# Interleukin-15 (IL-15) and Anti-C1q Antibodies as Serum Biomarkers for Ectopic Pregnancy and Missed Abortion

**DOI:** 10.1155/2013/637513

**Published:** 2013-01-17

**Authors:** Alexandros Daponte, Efthimios Deligeoroglou, Spyros Pournaras, Christos Hadjichristodoulou, Antonios Garas, Foteini Anastasiadou, Ioannis E. Messinis

**Affiliations:** ^1^Department of Obstetrics and Gynecology, University of Thessaly Medical School, University Hospital Larissa, Biopolis, 41110 Larissa, Greece; ^2^Division of Pediatric, Adolescent Gynecology and Reconstructive Surgery, 2nd Department of Obstetrics and Gynecology, Medical School, University of Athens and Aretaieion Hospital, Vasilisis Sofias 76, 11528 Athens, Greece; ^3^Department of Clinical Chemistry, University of Thessaly Medical School, University Hospital Larissa, Biopolis, 41110 Larissa, Greece; ^4^Department of Hygiene and Epidemiology, University of Thessaly Medical School, University Hospital Larissa, 6 Lapithon Street, Biopolis, 41221 Larissa, Greece

## Abstract

Given the present lack of clinically useful tests for the accurate diagnosis of ectopic pregnancy (EP), there is a need to select out those immunological factors measured in the maternal serum, as potential biomarkers. Our assumption was that C1q/anti-C1q antibody complexes and serum levels of interleukin-15 (IL-15) may play a role in differentiating abortions (MAs) and EPs and normal pregnancies. We assessed whether their measurement could set the diagnosis in a case control study at 6–8 weeks consisting of 60 women with failed early pregnancy (30 EPs, 30 MAs) and 33 women with intrauterine pregnancies. Normal pregnancies contain anti-C1q antibodies more frequently compared to women with failed pregnancies, the lowest levels being found in EPs, but this lacked statistical significance and anti-C1q could not serve as a marker. However EP pregnancies had elevated IL-15 levels that could statistically significantly differentiate them from MAs and IUPs. Furthermore, when assessing IL-15 for the clinically important differentiation between IUP and EP, we found at a cut-off of 16 pg/mL a negative predictive value of 99 with a sensitivity for diagnosing an EP of 92%. According to these results, serum IL-15 is a promising marker differentiating an MA from an EP.

## 1. Introduction

Unless a normal early intrauterine pregnancy (IUP) is visible by ultrasound, diagnosis can be a challenge [1–3]. When these patients present with pain and/or vaginal bleeding, the differential diagnosis between IUP and missed abortion (MA) or ectopic pregnancy (EP) is very difficult [4, 5]. The fear of intervening in the case of a desired pregnancy without certainty of diagnosis must be carefully weighed against the risk of misdiagnosing a missed abortion (MA) instead of an EP, due to the inherent danger to the mothers suffering an EP of tubal rupture and intraperitoneal haemorrhage.

Pregnancy is a natural example of an immune reaction occurring for a determined time period in the organism which opposes the rules of graft rejection [6]. The semi- or allogeneic fetal components in a normal pregnancy growing in the privileged site of uterus, not only escape maternal immune attack but also are supported by the maternal immune system [6].

Given the present lack of a clinically useful test for the accurate diagnosis of EP, there is a need to select out those immunological factors measured in the maternal serum, that are involved in the potentially disturbed maternal immune system's answer to the semiallogeneic conceptus in failed pregnancy cases and display the most promise to differentiate abortion (MA) and EP as potential biomarkers [3].

During implantation and early pregnancy, the immunological processes that take place within the uterus are to a great extent modulated by pro- and anti-inflammatory cytokines and their altered expression in the maternal serum may play a role in early pregnancy failure [7]. 

Successful pregnancy is considered a T helper 1 (Th1)-Th2 cooperation phenomenon, with a predominantly Th2-type lymphocyte response and specific cytokine production [8]. Th2 responses favour a cytokine milieu that promotes the induction of autoantibodies and several studies have attempted to link pregnancy failures and/or neonatal diseases with the presence of specific autoantibodies [9]. There has been an interest in the role played by anti-C1q antibodies, as these autoantibodies have been studied as prognosticators of disease flares and pregnancy outcomes in immune-mediated diseases such as systemic lupus erythematosus (SLE) [10] but not in women with missed abortions or ectopic pregnancies. 

Our assumption is that the formation of C1q/anti-C1q antibody complexes may also play a role in pregnancy failures such as MAs and EPs. We based our hypothesis on published reports (reviewed by Girardi et al. [9]) underlying the pivotal role played by C1q in promoting trophoblast invasion of deciduas, a crucial step in normal placental development. Thus, work on experimental models and C1q deficient mice has elegantly shown lack of C1q is characterized by poor trophoblast invasion and pregnancy failure [11]. 

As anti-C1q antibodies have not been tested as autoantibody markers in MA and EP, we assessed their presence and attempted to relate their appearance with the serum levels of interleukin-15 (IL-15). We have focused on the study of IL-15 as this cytokine is expressed by human placental tissue culture, its serum levels correlate with the duration of the pregnancy and it is maximally expressed during the implantation period in the deciduas [8, 12]. Notably, recurrent abortion cases are characterized by an upregulation of IL-15 expression in trophoblasts [13], suggesting that IL-15 can be a marker for pregnancy failure.

At 6–8 weeks gestational age the clinical differential diagnosis of a failed pregnancy is difficult due to uncertain dates of the last menstrual period or irregular cycles. We therefore set out to assess whether IL-15 serum measurement at 6–8 weeks could contribute to the differential diagnosis between failed pregnancies, whether EP or missed abortions (MA), and healthy intrauterine pregnancies (IUP). We also assessed the simultaneous presence of anti-C1q antibodies, as this could be a potential marker of the underlying immunopathological processes. 

## 2. Materials and Methods

### 2.1. Subjects

We performed a case control study consisting of 60 patients with failed early pregnancy presenting with mild abdominal pain or vaginal bleeding between 6–8 weeks of gestation, who were admitted to our tertiary centre between January 2009 and October 2010. Among the 60 cases included, 30 women had a ruptured EP, while 30 had MA. Serum samples were collected at the initial visit before treatment. If the clinician was unable to make a diagnosis on this first visit, the patient was admitted and followed up until a diagnosis of a viable intrauterine pregnancy or MA or EP was confirmed. All diagnoses were histologically confirmed. Serum beta HCG and IL-15 were measured in all 60 patients and in a group of 33 women with IUP between 6 and 8 weeks of gestation that served as a control group. EP, MA, and IUP women did not differ in terms of ethnicity (all Caucasian), maternal age (IUP: median 27 years (range 18–39); MA: median 35 years (range 21–45); EP median 32 years (range 26–44)), BMI (IUP: median 24 (range 19.9–31.2); MA: median 25.6 (range 20.7–35); EP: median 26.4 (range 21–34.5)), and smoking history.

The experimental testing complied with the principles laid down in the Declaration of Helsinki. All participating individuals gave informed consent to the work. The project was approved by the Larissa University Hospital Research Ethics Committee.

### 2.2. Beta HCG Measurement

Serum concentrations of beta human chorionic gonadotropin (HCG-*β*) were measured by an electrochemiluminescence immunoassay (ECLIA) intended for use on the automated analyzer Modular Analytics E170 (Roche Diagnostics GmbH, Mannheim, Germany). The results were expressed as mIU/mL and the lower limit of detection was <0.1 mIU/mL.

### 2.3. IL-15 ELISA Measurements

Serum samples were collected at the initial visit before treatment. All samples were processed by centrifuge (1,000 g for 15 minutes), and the supernatants were stored at −80°C until assayed. Serum concentrations of IL-15 were determined by quantitative sandwich ELISA (Quantikine human IL-15, R&D Systems, Minneapolis, MN, USA) according to the instructions of the manufacturer.: Quantikine human IL-15 is a quantitative sandwich enzyme immunoassay technique based on a monoclonal antibody specific for IL-15 which has been pre-coated onto a microplate. Testing steps have been followed in according to the protocols provided by the manufacturer. A standard curve based on the readings of 7 standards corresponding to 250 pg/mL, 125 pg/mL, 62.5 pg/mL, 31.2 pg/mL, 15.6 pg/mL, 7.8 pg/mL, and 3.9 pg/mL is used to calculate the test serum concentrations of IL-15. The 250 pg/mL standard serves as the high standard and the calibrator diluent serves as the zero standard (0 pg/mL). 

### 2.4. Anti-C1q Antibody Testing

IgG class anti-C1q antibodies have been tested by ELISA purchased from INOVA Diagnostics (San Diego, CA, USA). Testing has been carried out according to the manufacturer's instructions (http://www.inovadx.com/). Based on the provided leaflet, samples can be classified as negative (<20 AU/mL), weak positive (20–39 AU/mL, moderate positive (40–80 AU/mL), or strong positive (>80 AU/mL). 

### 2.5. Statistical Analysis

Skewed distributed variables are presented as median and interquartile range (IQR) (Table 1). 

**Figure 1:**
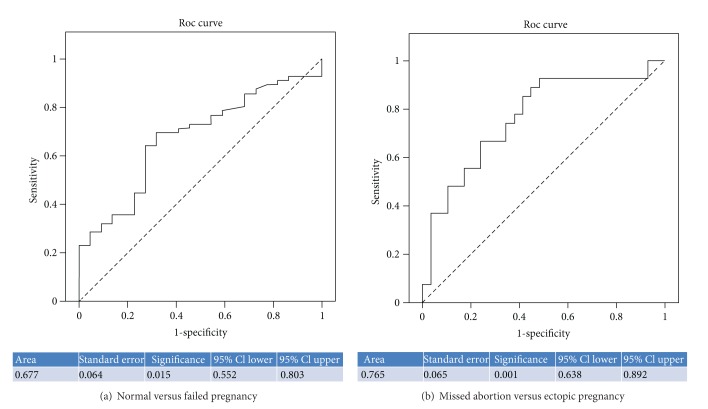
IL-15 ROC curves of normal versus failed pregnancy (a) and missed abortions versus ectopic pregnancies (b).

Analysis of variance was conducted in order to perform orthogonal contrasts (Helmert contrasts) comparing IUP versus MA and EP, as well as MA versus EP regarding IL-15. The optimal cut-off points for sensitivity and specificity were calculated by Receiver Operating Characteristics (ROC) curve analyses as the value with the maximum sensitivity plus specificity. According to the design ofthis study, the area under the curve (AUC) depicts the probability that the single value of IL-15 of a randomly selected patient with a normal pregnancy illustrated in Figure 1 (see below) will exceed that of a single value of a randomly selected patient with anabnormal pregnancy (EP or MA). Median values and interquartile range were calculated and sensitivity, specificity, positive predictive values (PPVs), and negative predictive values (NPVs) for EP were estimated (Table 2). 

The nonparametric Kruskal-Wallis test was used to identify differences on bhcg among EP, MA, and viable IUP. To perform pairwise comparisons between groups Mann-Whitney test was conducted determining as critical value for significance *P* = 0.0167 after using Bonferroni correction.

Basic demographic characteristics such as age, BMI were compared using Kruskal-Wallis.

Spearman's rank correlation coefficient (*ρ*) was used to explore the relationship between beta HCG with the other measures. All statistical analyses were performed in SPSS 15 statistical software (Chicago, IL, USA). A *P* value less than 0.05 was considered statistically significant. 

## 3. Results

### 3.1. Diagnostic Accuracy of Anti-C1q Antibodies

Strong (>80 AU/mL) anti-C1q antibody reactivity was found in 4/30, 4/30, and 6/33 women with EP, MA, and IUP, respectively (not significant). Moderate or strong anti-C1q antibody reactivity (>40 AU/mL) was present in 10/30 women with EP, 8/30 women with MA, and 14/33 women with IUP (not significant).

As our data are not normally distributed, the nonparametric Kruskal-Wallis test was used to identify any difference on anti-C1q among groups of pregnancy outcome. However, in order to find out which groups differ, a Mann-Whitney test was performed conducting three pairwise comparisons. Additionally, using Bonferroni correction, as critical value of significance for each (Mann-Whitney) test was determined the value 0.0167. Therefore anti-C1q antibody levels are not helpful in differentiating between the three groups since as shown in Table 1 there is no *P* value <0.167.

### 3.2. Diagnostic Accuracy of IL-15

IL-15 concentrations were significantly higher in 60 pregnancy failures (median 19.86, IQ range 16.04–26.97 pg/mL) compared to women with a viable IUP (median 15.06, IQ range 12.74–21.41 pg/mL) and this was highly significant (*P* < 0.001, Table 2). 

This is mainly the result of the fact that IL-15 concentrations were significantly higher in women with EP (*n* = 30, median 24.9 pg/mL) compared to patients with IUP (*n* = 33, 15.06 pg/mL), *P* < 0.001 (Table 2). Additionally, IL-15 had the ability to discriminate an EP from MA (*P* = 0.015) (Table 2). The corresponding ROC analyses were calculated and plotted for the diagnostic accuracy of serum IL-15 concentration to discriminate between the groups (AUCs in Table 2) (Figure 1).

IL-15 values were plotted in ROC curves in order to further evaluate their diagnostic accuracy for the diagnosis of healthy IUP and for accurate discrimination of an EP from an MA. All AUCs are shown in Table 2. IL-15 showed high diagnostic accuracy for the discrimination of a viable IUP from an EP with an area under the curve (AUC) 0.818, (Table 2, Figure 1) and at the threshold of 16.1 pg/mL, had a sensitivity of 92% and a specificity of 68%, and a clinically important negative predictive value (NPV) of 0.999 for diagnosing a normal from an EP. According to this, if the IL-15 concentration is less than 16 pg/mL it is highly unlikely that the pregnant woman suffers from an EP.

### 3.3. IL-15 Maternal Serum Concentration Relationship to Beta HCG

Since IL-15 was showing to be a promising biomarker we further analysed its serum concentration relationship with bHCG. IUPs had a median bHCG concentration of 59,668 mIU/mL (40,156–87,906 mIU/mL), while MAs had a median of 3000 mIU/mL (1447–5500 mIU/mL) and EPs a median of 1828 mIU/mL with an IQR 1147–2790 mIU/mL (Kruskal-Wallis test, *P* < 0.001).

In order to further explore pairwise comparisons between groups, we conducted Mann-Whitney test using the Bonferroni correction. It was identified that IUPs have significantly higher values of beta HCG compared to MAs and then to EPs, (*P* < 0.001). Between MAs and EPs there was no statistically significant difference (*P* = 0.115).

Spearman's rank correlation coefficient (*ρ*) between beta HCG and IL-15 in IUPs was 0.033 (*P* = 0.884), in MAs −0.141 (*P* = 0.521) and in EP 0.115 (*P* = 0.585) demonstrating that there is no correlation between IL-15 and bHCG values in all three study groups.

## 4. Discussion

Currently, there is no stand-alone diagnostic biomarker for tubal ectopic pregnancy that has been adequately tested and yields satisfactory results. The clinical end-point of this study was the potential identification of EP cases by a single serum measurement of IL-15. In a similar context, we tested for anti-C1q antibodies, as a potential marker of pregnancy failure.

Much to our surprise, we have found that serum samples from women with normal pregnancies contain IgG anti-C1q antibodies more frequently compared to those with failed pregnancies, the lowest level of detectable antibodies being found in women with EP. In our study, 18% (6/33) of women with IUP have shown strong IgG anti-C1q antibody reactivity. This percentage is comparable to that of 20% recently reported in a study testing 30 women with normal pregnancies [14]. Antibodies to C1q are found in SLE (including pregnant women) and a variety of other autoimmune and nonautoimmune conditions, including healthy individuals, and their role in the induction of pathological processes remains elusive [10, 15–20]. Stoyanova et al. [14] have suggested that the presence of anti-C1q antibodies in women with normal pregnancies could be associated with their physiological and pregnancy state or their current hormonal status. This hypothesis needs to be explored further, as our data argue in support of the notion that failed pregnancies and in particular those with EPs are immunologically privileged in respect to the loss of immunological tolerance to C1q. 

Nevertheless, the great majority of women, irrespectively of whether they have failed or normal pregnancies, lack autoantibodies against C1q and this needs to be taken into account when exploring the immunobiology of pregnancy in relation to humoral immunity specific for C1q. 

Biomarkers which reflect the viability of a pregnancy may be higher in normal IUPs but may not differentiate an abnormal pregnancy in the uterus (miscarriage) versus an abnormal pregnancy in the fallopian tube (EP). Markers reflecting the location of the pregnancy, rather than viability, may therefore be able to differentiate between the two types of nonviable conceptuses: a miscarriage and an EP. 

Our analysis has shown that EP pregnancies had increased IL-15 levels that could statistically significantly differentiate them from MAs and IUPs. Furthermore, when assessing IL-15 for the clinically important differentiation between IUP and EP, we found at a cut-off of 16 pg/mL, a negative predictive value of 99 (AUC of 0.818) (Table 2) with a sensitivity for diagnosing an EP of 92%. According to these results, serum IL-15 is a marker that can differentiate an MA from an EP (AUC of 0.753). This comes as no surprise as Toth et al. [13] reported that IL-15 expression is upregulated in placental tissue of disturbed human first trimester pregnancy and trophoblast cells were detected as a main source for IL-15 in women with recurrent miscarriages. The trophoblast invasion in ectopic pregnancy is different from normal pregnancies [21, 22] and this may explain differences related to IL-15 tissue expression and circulating levels of this cytokine [13]. Trophoblast infiltrating the tube or the peripheral NK cells can be the source of the increased levels of IL-15 in EPs compared to IUPs, but this needs to be explored further. This is in accordance to our finding of increased IL- 15 levels in EP which due to its protective effect might explain the surviving of trophoblasts while penetrating the tubal wall. However it must be emphasized that the aim of the present study was not to explore whether increase in IL-15 directly or indirectly relates to the development of EP and MA. Thus, it is uncertain whether the high levels of IL-15 in pregnancy failure are the consequence of the pathological processes that take place or whether their increase participates in the induction of EP, with increased serum levels simply reflecting the involvement of IL-15 in the pathogenesis of the disease. The pathophysiological significance of our observations can only be studied through serial measurements and further tissue expression assessment of these markers in a larger prospective study.

Our group performed a number of studies reporting that maternal serum angiogenic factors vascular endothelial growth factor receptor [VEGF-R1 (flt-1)], VEGF, angiopoetin (ANG-)1 and ANG-2 are potential markers of failed pregnancies—MA and EP, as their levels are significantly decreased in early pregnancy failure (MA or EP) at 6–8 weeks of gestation compared to those found in healthy intrauterine pregnancies [3, 23, 24]. 

Therefore IL-15 could be an ideal additional biomarker to the already reported angiogenic factors in a multimarker diagnostic kit possibly reflecting different pathogenic mechanisms of pregnancy failure. In our series of failed pregnancies the IL-15 values did not correlate with beta HCG values raising the expectation that these parameters could be HCG-independent biomarkers, which could supplement transvaginal ultrasound and other biomarkers in a future multimarker algorithm. 

Of relevance, the median beta HCG value in our EPs was at 1828 mIU/mL, which is a level with a known difficulty in identifying a gestational sac by ultrasound that would exclude an EP.

The possible clinical implications of these findings are that women with threatened abortions and no visible gestational sac could be scheduled for regular follow-up antenatal visits and repeat ultrasounds if the IL-15 level is lower than 16 pg/mL as it has an excellent NPV for EP. Furthermore it could be argued that these women may not need to be admitted as possible pregnancy failures with obvious cost benefits.

Our results on IL-15 must be tested in a longitudinal or retrospective cohort study and validated in a larger prospective study to determine its potential clinical value, ideally in a prospective multicenter study. We hope our findings may contribute to the research that is underway to both identify novel biomarkers and combine new and existing markers into a multiple marker test with the goal of accurately identifying ectopic pregnancies.

## Figures and Tables

**Table 1:** Anti-C1q antibody concentrations in 60 women with failed pregnancies, including 30 women with ectopic pregnancy (EP) and missed abortion (MA) and 33 women with intrauterine pregnancy (IUP). The nonparametric Kruskal-Wallis test was used to identify differences among EP, MA, and viable IUP. To perform pairwise comparisons between groups Mann-Whitney test was conducted determining as critical value for significance *P* = 0.0167 after using Bonferroni correction which determined as critical value of significance for each (Mann-Whitney) test the value 0.0167. Therefore anti-C1q antibody levels are not helpful in differentiating between the three groups since as shown in Table 1 there is no *P* value <0.167.

		Failed pregnancies (MA and EP)			Normal	*P* value
Anti-C1q	Median	30.92			38.95	0.018*
	IQR	17.89–40.79			28.95–59.01	

		Pregnancy outcome				
		IUP	MA	EP		

Anti-C1q	Median	38.95	31.97	18.82		0.045**
	IQR	28.95–59.01	23.95–39.47	16.32–40.79		

*Mann-Whitney test.

**Kruskal-Wallis test.

**Table 2:** IL-15 concentrations in 60 women with failed pregnancies, including 30 women with ectopic pregnancy (EP) and missed abortion (MA) and 33 women with intrauterine pregnancy (IUP). Median, interquartile range (IR) in pg/mL, and the Mann-Whitney *P* value are presented. Additionally sensitivity, specificity, positive predictive value (PPV), and negative predictive value (NPP) were calculated and IL-15 values were plotted in ROC curves in order to further evaluate their diagnostic accuracy for the diagnosis of healthy IUP and for accurate discrimination of an EP from a MA. All AUCs are shown.

IL-15 Serum levels	Median (interquartile range) Mann Whitney *P *	Sensitivity	Specificity	Cut-off point	Direction	AUC	*P* value (ROC curve)	PPV	NPV
Failed versus normal	19.86 (16.04–26.97) 15.06 (12.74–21.41) *P* < 0.001	0.750	0.682	16.1	Upper	0.725	0.002	0.310	0.935

Abortion versus normal	16.63 (14.56–21.37) 15.06 (12.74–21.41) *P* < 0.001	0.857	0.364	14.0	Upper	0.605	0.207	0.192	0.935

Ectopic versus normal	24.9 (18.59–29.81) 15.06 (12.74–21.41) *P* < 0.001	0.917	0.682	16.1	Upper	0.818	<0.001	0.028	0.999

Ectopic versus abortion	24.9 (18.59–29.81) 16.63 (14.56–21.37) *P* = 0.015	0.861	0.571	16.9	Upper	0.753	0.001	0.118	0.984

## Abbreviations

AUC: Area under the curve
EP: Ectopic pregnancy
IL-15: Interleukin 15
IUP: Intrauterine pregnancy
MA: Missed abortion.
